# A Fear of COVID-19 and PTSD Symptoms in Pathological Personality: The Mediating Effect of Dissociation and Emotion Dysregulation

**DOI:** 10.3389/fpsyt.2021.590021

**Published:** 2021-03-23

**Authors:** Patrizia Velotti, Claudia Civilla, Guyonne Rogier, Sara Beomonte Zobel

**Affiliations:** Department of Dynamic and Clinical Psychology, Faculty of Medicine and Psychology, Sapienza University of Rome, Rome, Italy

**Keywords:** fear of COVID-19, posttraumatic stress disorder, emotion dysregulation, pathological personality, longitudinal, dissociation

## Abstract

**Background:** The 2019 coronavirus disease (COVID-19) outbreak is currently putting a strain on the mental health resilience of the world's population. Specifically, it is likely to elicit an intense response to fear and to act as a risk factor for the onset of posttraumatic stress disorder (PTSD). Some individuals may be more at risk than others, with pathological personality variables being a potential candidate as a central vulnerability factor. In addition, the pathways that lead the pathological personality to PTSD and intense fear responses to COVID-19 are likely to be explained by poor emotion regulation capacities, as well as by dissociative mechanisms.

**Aims:** This study aimed to shed light on vulnerability factors that may account for the onset of PTSD and intense responses of fear in response to COVID-19 outbreak and to test the mediating role of emotion dysregulation and dissociation proneness in these pathways.

**Methods:** We used a longitudinal design of research administered to a sample of community individuals (*N* = 308; mean_age_ = 35.31, SD = 13.91; 22.7% were male). Moreover, we used self-report questionnaires to measure pathological personality, emotion regulation capacities, dissociative proneness at the beginning of the lockdown, and PTSD symptoms and fear of COVID-19 at the end of the Italian lockdown (from March 9 to May 18, 2020). Structural equation modeling was used to test the hypotheses.

**Results:** We found that pathological personality levels longitudinally predicted PTSD and fear of COVID-19 levels. Moreover, the associations between emotion dysregulation and dissociation were shown to significantly and totally mediate the relationship between pathological personality and PTSD, whereas no significant mediation effects were observed in relation to fear of COVID-19.

**Conclusions:** Individuals with pathological personality traits may be more vulnerable to the onset of negative psychological consequences related to COVID-19 outbreak, such as PTSD symptomatology and fear levels. Emotion regulation capacities appear to be relevant targets of interventions for PTSD symptomatology. Future research should explore the mediating variables linking pathological personality to intense fear responses to COVID-19.

## Introduction

According to the World Health Organization (1), the new coronavirus disease (COVID-19), which appeared at the end of the 2019, is responsible for more than 1,633,941 deaths and 73,453,006 confirmed cases (December 15, 2020). Most nations have been forced to implement restrictive rules to protect citizens' health, such as “stay-home” indications, preventing individuals from moving freely, which often implied the impossibility of physically interacting with their significant others. In particular in Italy, given the rapid spread of the virus over the entire peninsula, a total lockdown was established (with the closure of commercial activities and services and ban on free movement) from March 9 to May 18, 2020. It seems reasonable to suggest that this situation may have induced relational and social difficulties (2). Moreover, this serious scenario could also be worsened by an amplification of fear (3), by media or social networks, spreading panic or fake news, or by false-positive diagnosis (for instance, in India, a family man committed suicide to protect his family following a false-positive diagnosis of COVID-19). Fear can be dangerous, especially for subjects with previous psychopathological vulnerability, such as pathological personality traits: studies examining basic biological mechanisms support the idea that emotion dysregulation increases fear levels (4), through the connection between emotional dysregulation and lack of tolerance. In addition, the relationship between anxious personality levels and the tendency to generalize fear in new situations (i.e., toward stimuli with perceptual or conceptual similarities to an original fearing stimulus) has been confirmed by different researches (5–8).

Some authors (9) have reviewed the hypothesis that fear experienced during a traumatic event, followed by repeated memories related to it, may lead to a sensitization of the response to fear with a consequent heightened psychophysiological reactivity. In fact, recent studies conducted during the COVID-19 outbreak pointed out the psychological consequences of the pandemic, such as emotional disturbances, depression, anger, insomnia, and emotional exhaustion triggered by fear and isolation (10). In some cases, the pandemic was experienced as a real traumatic event, and some studies have found that it led to the development of posttraumatic stress disorder (PTSD) in both Italian and world population (11–14) as other dramatic events studied before (15, 16). PTSD, diagnosed for the first time in war survivors and known as “battle fatigue,” has been widely studied in all populations who have experienced traumatic events, such as natural disasters, terrorist attacks, car accidents, sexual abuse, and death of loved ones (17).

PTSD is classified within the section Disorders Related to Traumatic and Stressful Events (American Psychiatric Association, 2013), and its onset occurs after the exposure to a traumatic or stressful event. PTSD consists of symptomatologic manifestations, such as dissociative reactions, recurrent intrusive memories, avoidance of trauma-related stimuli, and negative alteration of emotions associated with the event. Recently, some authors have hypothesized a correlation between PTSD and pathological personality, intended as a disturbance that fundamentally involves dysregulation and/or distortions in both proximal and internal interpersonal situations (18). An increased interest in the study of pathological personality allowed the creation of diagnostic manuals over the years, aiming to describe the plurality of personality disorders and their characteristic features. The frequency of personality disorders in general adult population has been estimated to be approximately 12.16% and to be three times higher among clinical populations (19). Personality disorders greatly affect the costs of public health, as individuals who suffer from these disorders often show great difficulties in their social, work, and interpersonal functioning (20). These costs are likely to increase when worldwide population is struggling with a severe blow, such as the COVID-19 outbreak.

A group of researchers (21) investigating the relationship between PTSD, posttraumatic beliefs, and personality traits in subjects who had experienced disasters found that pathological personality was positively related to PTSD symptoms. In particular, individuals with PTSD showed high levels of mood instability and grandiosity traits. Another line of research (22–24) investigated the association between borderline personality disorder (BPD) and PTSD and hypothesized a common etiology that focuses on the associations between childhood trauma, particularly childhood sexual abuse, and both BPD and PTSD. The results showed that some key characteristics of the two disorders, such as affective instability, cognitive/perceptual disorders, and interpersonal dysfunctions, overlapped (25–27). James et al. (28) observed that a group of veterans with PTSD showed higher levels of pathological personality [measured using the Personality Inventory for *Diagnostic and Statistical Manual of Mental Disorders, Fifth Edition* (*DSM-5*)] than another group of veterans without PTSD. Specifically, the most problematic domains were those related to psychoticism, detachment, disinhibition, and negative affection. According to the authors, this study emphasizes the importance of pathological personality in PTSD, paving the way for new research aiming at evaluating the role of maladaptive personality traits in PTSD comorbidities.

Currently, the best way to prevent COVID-19 is to avoid being exposed to the virus. However, a scenario characterized by the need to wear gloves and masks and the lack of any effective available therapy may lead to an uncontrolled spread of fear. A fear response, as a basic physiological condition, occurs when there is a threat, whether real or perceived. Its role is to prepare the body to respond to this threat. However, if there is a dysfunction in the processing of fear, it can lead to the development of psychiatric symptomatology (29). LeDoux (30, 31), who has extensively studied fear and the role of the amygdala, has identified three ways by which individuals react to frightening stimuli. The fastest circuit is the “primitive” one, which puts in place an immediate fight-and-flight response; the “rational” circuit is slower and allows the individual to consider the situation in a more realistic way; the “reflective” circuit is characterized by the awareness of being afraid. The primitive circuit guarantees survival; however, if the frightening stimulus is far enough, the rational circuit is activated, helping to rationalize what is happening. The rational circuit is not as immediate as the primitive circuit and does not always work properly, which is why emotions sometimes take over. Another important contribution comes from Porges' polyvagal theory (32). The author asserted that three levels of activation exist as a function of environmental conditions: a “safe environmental situation,” in which social interaction acts as a mediator of autonomic modulation and in which the systems of attachment and socialization are facilitated; a “situation of insecure environment,” which causes active avoidance reactions that provide the possibility to attack or escape in an adaptive way; a “life-threatening situation,” which is a condition wherein the threat is so overwhelming that the reactions activated are mostly characterized by passive avoidance, such as freezing, dissociation, tonic immobility, and feigned death (32). All these studies converge on the idea that dissociation can be a form of emotion regulation that is used to cope with various stressful situations (33). Moreover, several authors have conceptualized dissociation as an experiential avoidance strategy that aims to decrease awareness or the processing of painful affects (34); therefore, it confirms it is deeply connected to emotion regulation abilities.

## Emotion Dysregulation and Dissociation in PTSD

In the third level of activation of Porges' polyvagal theory, the individual implements a dysfunctional modality to regulate emotional arousal and may use dissociation. The proposed *DSM-5* criteria for PTSD conceptualize the disorder as a mere response to fear and include dysregulation of negative emotions, including anger, guilt and shame, and dissociation. Dissociation plays an important role in PTSD, inducing disruptions in the integrated functions of memory, identity, and perception of oneself and the environment (26, 33, 35).

Furthermore, research has shown that poor emotion regulation exacerbates PTSD symptoms (36), suggesting that emotion dysregulation is a mechanism that emphasizes or makes them chronic (33). According to Gratz and Roemer (37), emotion regulation in adulthood can be considered as a multidimensional construct that includes awareness, understanding, and acceptance of emotions. It promotes the ability to control impulsive behaviors and to implement goal-directed behaviors when experiencing negative emotions, guaranteeing the use of situational strategies to modulate the intensity and the duration of emotional responses, instead of completely eliminating them (38).

The difficulty in regulating emotions is connected to a series of maladaptive behaviors (39), such as addiction, promiscuous sexual behavior, and self-harm (40–44), as well as several psychopathologies (45), such as BPD, PTSD, or generalized anxiety disorder. Studies on patients with BPD have shown that emotion dysregulation seems to develop simultaneously with dissociative features (46). In addition, Briere (47) conducted a study on traumatized patients and found a relationship between PTSD, emotion regulation, and dissociation. Even though these results suggest a link between emotion regulation and PTSD, to date few studies have examined the interplay between emotion dysregulation and dissociation in PTSD (48).

## The Current Study

As the variables of fear, personality, emotional dysregulation, and PTSD are extremely interrelated with each other, it seemed important to us to study them jointly during the COVID-19 pandemic in order to better understand the mechanisms that bind them. Thus, the purpose of this study was to investigate the population subjected to “stay-home” measures related to the COVID-19 outbreak, and the hypothesis is therefore that personality pathology severity predicts PTSD and fear of COVID-19 and that these relationships may be mediated by emotion dysregulation and dissociation.

Therefore, beyond the importance of examining this relationship within the context of the pandemic, this study aimed to provide useful evidence to fill the gap in literature on the interplay between personality, PTSD, and emotion dysregulation.

## Methods

### Participants and Procedure

All participants were recruited through an online survey distributed 3 days after the beginning of the lockdown (Time 1). A presentation letter at the beginning of the survey explained the aims and scopes of the study and illustrated information regarding anonymity and privacy. Then, participants were invited to provide an informed written consent and to fill in a battery of self-report questionnaires. Lastly, an email was sent to each participant 3 days before the end of the lockdown (Time 2), asking to complete the procedure through the answering of additional self-report questionnaires. The whole procedure was approved by the ethics committee of the University of Rome, Sapienza (N. 356/20).

At the survey at Time 1, 1,323 subjects responded (mean_age_ = 35.38, SD = 14.08); 23% of the sample were male; 52.3% had achieved a level of education higher than high school diploma, but nearly half of the sample reported having an income of <€36,000 per year. As for romantic relationships, 33.7% said they were not involved in any relationship, and 26.5% of them reported having one or more children. For the purpose of the study, only participants who completed the two batteries of self-report questionnaires (at Time 1 and Time 2) were considered. The final sample consisted of 308 adults (mean_age_ = 35.31, SD = 13.91; 22.7% males). Among them, 44.3% obtained a college degree, and nearly half (47%) had an income per year inferior to €36,000. In addition, most were not involved in any romantic relationship (23%), and only 24.4% of the sample reported to have children.

## Measures

The battery of self-report questionnaires administered at Time 1 consisted of the following:

- a questionnaire asking for demographic information such as age, gender, and economical incomes;
- emotion dysregulation levels were measured through the *Difficulties in Emotion Regulation Scale* [DERS; (37, 49)]. This is a self-report questionnaire of 36 items asking the participant to answer on a Likert-type scale ranging from 1 (never) to 5 (always). It provides not only a total score but also subscores corresponding to the six dimensions of emotional dysregulation evaluated by the instrument, namely, (i) nonacceptance (the difficulty to accept negative emotions in a nonjudgmental way), (ii) goals (the deficit in the ability to pursue goal-directed behavior when experiencing negative emotions), (iii) impulse (the tendency to act in a rush when experiencing negative emotions), (iv) awareness (the lack of awareness of one's negative emotional states), (v) clarity (the difficulty to discriminate between negative emotional states) and strategies (the perceived lack of available and effective emotion regulation strategies to regulate negative emotions);
- dissociative experiences were evaluated throughout the Dissociative Experiences Scale II [DES-II; (50, 51)]. This instrument is a 28-item self-report questionnaire asking the participant to evaluate the frequency by which some dissociative experiences occur in his life on an 11-point Likert-type scale ranging from 0% (never) to 100% (always). The instrument provides a total score and two scores corresponding to the two subscales of the questionnaire being detachment and compartmentalization;
- pathological personality was assessed with the Pathological Inventory for *DSM-5*, short version [PID-5; (52, 53)]. This self-report questionnaire consists of 25 items asking the participant to answer on a 4-point Likert-type scale ranging from 0 (always or often false) to 3 (always or often true). The instrument provides five scores corresponding to the main five pathological domains of personality identified by *DSM-5*. These are negative affect, disinhibition, antagonism, detachment, and psychoticism.

At Time 2, participants were asked to fulfill other self-report questionnaires evaluating the following variables:

- PTSD symptom severity, assessed throughout the National Stressful Events Survey PTSD Short Scale (NSESSS), a 9-item instrument developed by LeBeau et al. (54). It asks to answer to each item on a 5-point Likert-type scale, with answers potentially ranging from 0 (not at all) to 4 (extremely). This provides a total score assessing the severity of PTSD symptoms. We adapted the version of this instrument asking the participant to evaluate only stressful events related to the emergency situation derived from the onset of COVID-19, specifying in the questionnaire submission: “In the past 2 weeks, how disturbed you have been with each of the following problems that began or worsened following an extremely stressful event or experience related to the COVID-19 pandemic?”
- fear of COVID-19, measured throughout the Fear of COVID-19 Scale [FCV-19S; (55, 56)]. This self-report questionnaire asks the participant to indicate the intensity of seven experiences related to the experience of fear linked to COVID-19, answering on a 5-point Likert type scale. The scale was constructed from an extensive literature review of all general fear scales tested across different populations and diseases. To date, the instrument has been translated and validated in more than 19 languages, and its predictive power on anxiety, health anxiety, and PTSD symptoms has been confirmed by several studies (57).

## Statistical Analyses

To reach the aims of the study, we first explored bivariate correlations between all variables included in the study, throughout the calculation of *r* Pearson coefficients. Then, we designed and tested a structural equation modeling. First, latent variables were created using manifest variables. Specifically, pathological personality latent variable was the result of the convergence of the scores obtained on the five subscales of the PID-5. Emotion dysregulation latent variables resulted from the convergence of the six scores obtained on the six subscales of the DERS. Similarly, dissociation latent variable was created using the scores obtained on the subscales of DES-II. Finally, fear of COVID-19 and PTSD symptomatology were two latent variables created using the scores obtained on the items of FCV-19S scale and NSESSS, respectively.

The structural model has been designed specifying paths between independent variable (pathological personality), mediators (dissociation and emotion dysregulation), and outcomes (fear of COVID-19 and PTSD symptoms). As age resulted to be significantly correlated with our outcomes, we decided to include it as a covariate in the structural model.

To design and test our model, we used the lavaan package of the R software for Mac. The method used evaluates the consistency of a dataset with a model previously defined throughout the robust maximum likelihood method of estimation. Results brought by these statistical analyses are examined using several goodness-of-fit indexes, such as root mean square error of approximation (RMSEA), Tucker–Lewis coefficient (TLI), and comparative fit index (CFI). A 0.05 < RMSEA > 0.08 (58) and both TLI and CFI being > 0.90 (59) are generally interpreted as an adequate fit. In addition, we examined the lower and upper boundaries of the 90% confidence interval for RMSEA, with an upper boundary of more than 0.10, indicating that the model should be rejected (58).

## Results

### Correlations Between Variables

We calculated the *r* Pearson correlations between all variables involved in the study at Time 1. The results are fully displayed in Table 1.

**Table 1:** Correlations between main variables of the study.

	**PID NA**	**PID DET**	**PID ANT**	**PID DIS**	**PID PSY**	**DERS**	**DESdet**	**DEScomp**	**PTSD**	**FEAR**	**Age**
PID NA	–										
PID DET	0.53^**^	–									
PID ANT	0.27^**^	0.44^**^	–								
PID DIS	0.39^**^	0.41^**^	0.29^**^	–							
PID PSY	0.60^**^	0.58^**^	0.37^**^	0.42^**^	–						
DERS	0.52^**^	0.56^**^	0.29^**^	0.36^**^	0.54^**^	–					
DESdet	0.31^**^	0.34^**^	0.21^**^	0.35^**^	0.44^**^	0.36^**^	–				
DEScomp	0.35^**^	0.38^**^	0.24^**^	0.35^**^	0.50^**^	0.39^**^	0.75^**^	–			
PTSD	0.42^**^	0.40^**^	0.21^**^	0.30^**^	0.44^**^	0.51^**^	0.48^**^	0.55^**^	–		
FEAR	−0.28^**^	−0.15^*^	−0.08	−0.01	−0.25^**^	−0.29^**^	−0.11	−0.21^**^	0.22^**^	–	
Age	−0.28^**^	−0.15^*^	−0.08	−0.01	−0.25^**^	−0.29^**^	−0.11	−0.21^**^	−0.22^**^	−0.11^*^	–

*PID, Pathological Inventory for DSM-5; NA, Negative Affect; DET, Detachment; DIS, Disinhibition; PSY, Psychoticism; DERS, Difficulties in Emotion Regulation Scale; DEScomp, Dissociative Experience Scale 2nd Version Compartmentalization; DESdet, Dissociative Experience Scale 2nd Version Detachment scale; PTSD, Post Traumatic Stress Symptoms; FEAR, Fear of COVID-19*;

** *p < 0.001*;

* *p < 0.05*.

## Structural Equation Model

**Figure 1:**
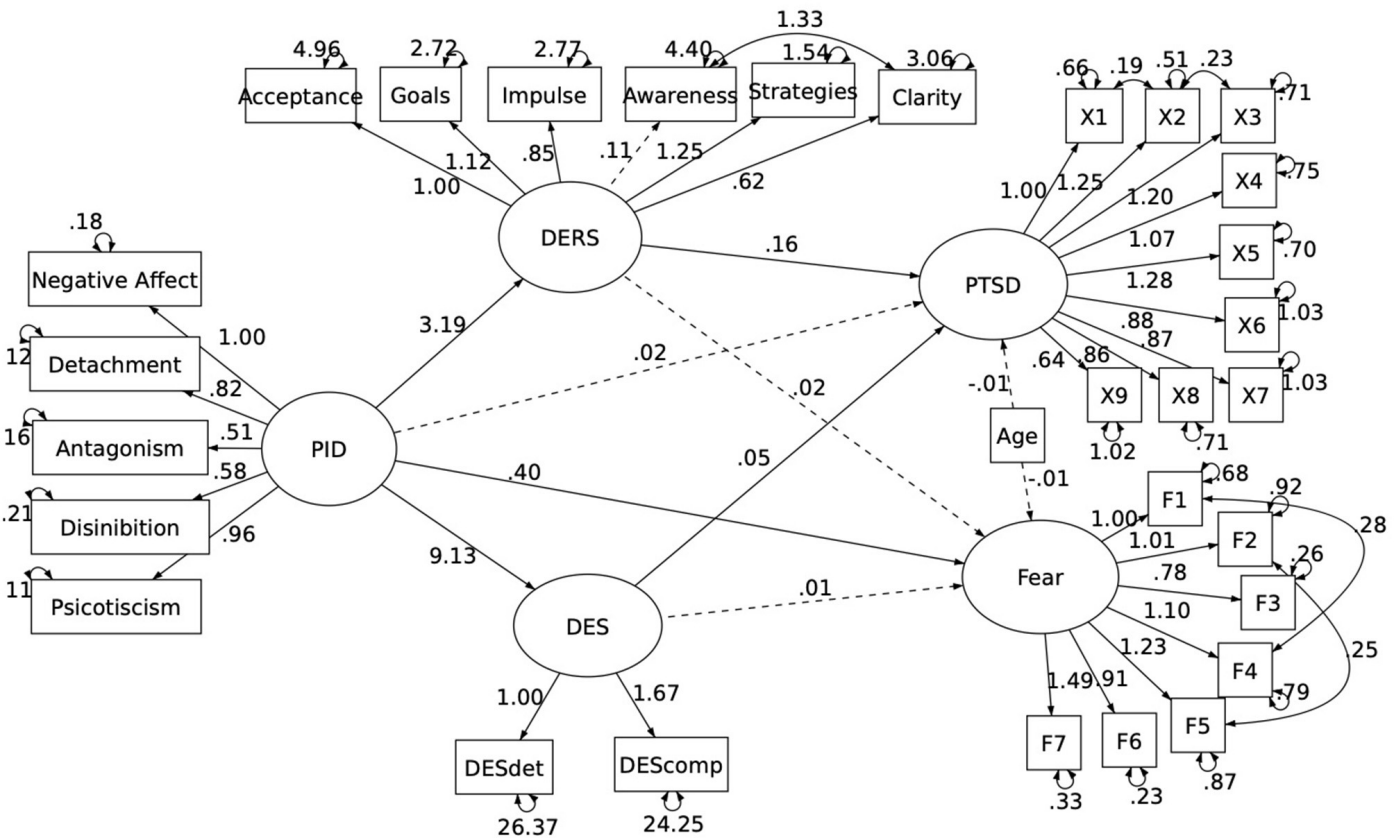
Final Model illustrating the longitudinal relationship between pathological personality and both PTSD and Fear of Covid.

The test of the first model brought an acceptable fit according the RMSEA index [0.069; 90% confidence interval (CI) (0.064–0.075)], but the CFI index was considered below the acceptable cutoff (0.87). Thus, we respecified the model using the modification indexes suggested by the software. In particular, we added the estimation of some parameters, including the covariance between residual errors between the PTSD (Item 2 and Items 1 and 3) and FEAR items (2 and 5; 1 and 4), and the clarity and awareness dimension of the emotion dysregulation variables. These respecifications allowed us to reach an acceptable fit for the model on both the RMSEA [0.060; 90% CI (0.054–0.066)] and CFI index (0.90). The final model, as illustrated in Figure 1, indicates that pathological personality positively and significantly predicted the level of fear of COVID-19. However, this effect was not mediated by emotion dysregulation (ß < 0.05; *p* = 0.616) nor dissociative features (ß < 0.02; *p* = 0.745). In contrast, the relationship between pathological personality and PTSD symptoms was totally mediated by both emotion dysregulation (ß < 0.51; *p* .001) and dissociation (ß < 0.45; *p* .001).

## Discussion

In the present study, we tried to understand the relationship between pathological personality, PTSD, and fear of COVID-19. Furthermore, we investigated whether and how emotion dysregulation and dissociation mediate the relationship between these variables.

First, the results confirmed a direct relationship between pathological personality and fear of COVID-19, which is consistent with results brought by literature that hypothesized and observed among clinical samples that individuals with an anxious personality showed higher fear scores (5).

Second, the results confirmed a direct relationship between pathological personality and PTSD, which is consistent with our hypotheses and the results brought by literature. For instance, Reis et al. (21), in a study conducted on individuals who experienced disasters, found a positive and significant correlation between pathological personality and PTSD levels.

For both pathways, we assumed the existence of intervening mediators, such as emotion dysregulation and dissociation. Regarding the relationship between pathological personality and fear of COVID-19, our results did not confirm that dissociation and emotion dysregulation were involved in the relationship between the two variables. Thus, we think that other intervening mediators, which are not examined in the study, may better explain this relationship. For example, Reis et al. (21) underlined the role of specific variables, such as grandiosity and unstable mood. On the contrary, in the relationship between pathological personality and PTSD, the results highlight the important role of emotion dysregulation and dissociation, supporting the results of previous studies (47, 48). Indeed, many authors have studied PTSD and its comorbidity with personality disorders: for instance, Briere (47) and other researchers have become interested in the role played by dissociation and dysregulation of emotions in PTSD and fear, whereas Van DiJke et al. (60) assert that exposure to trauma or a series of trauma leads to the development of posttraumatic symptoms and development of personality disorders. Furthermore, they hypothesized that in the event that elicits a posttraumatic complex stress disorder, some factors, such as poor emotion regulation and dissociative characteristics, can worsen the clinical picture. Thus, it can be deduced from the many studies that highlighted the strong link between pathological traits and PTSD. Furthermore, emotion dysregulation has been linked both to the development or worsening of PTSD and to the severity of other psychopathologies related to trauma. Among other things, more and more research (9, 61) supports the existence of a dissociative subtype of PTSD that has been included in the fifth edition of the *DSM* (62).

## Limitations and Future Directions

Our study has objective limitations that must be considered. Given social distancing rules and the inability to conduct research in any other way, we used nonprospective tools to collect data, specifically self-reports that can be filled online. Although they showed good reliability and internal consistency and provide a valid, easy, and fast assistance in the administration of diagnostic tools, self-report instruments are limiting for various reasons. First, the possibility that the constructs investigated are easily identifiable can lead to the phenomenon of social desirability, the creation of prejudices, and therefore the falsification of answers. For this reason, the use of implicit measures to assess personality could be an excellent research starting point for the future (63). Another aspect to be taken into account is the possibility of overlooked latent variables that could have had an effect on the relationships identified. Nevertheless, what has been said can be a starting point to deepen these aspects in future research and possibly compare the results. We can therefore assert that this research may allow an opening to new considerations both on personality disorders and on the factors involved in the development of trauma: it could be very interesting to investigate the personality more broadly, perhaps making use of projective instruments, in relation to COVID trauma to identify protective factors or vulnerabilities that could promote or worsen traumatic adaptation. In addition, our study highlights that the pandemic experience (i.e., staying home and related deprivations) may have led to the onset of difficulties in general population, but even more in vulnerable individuals with pathological personality traits (61, 64–66).

Concerning possible clinical implications, this study sheds light on which could be the most relevant aspects to focus on for intervention: reading COVID-related distress in terms of PTSD could be useful for formulating specific therapeutic interventions, especially with individuals with preexisting psychopathology. The research results could therefore be used in diagnostics and exploration, as well as open new directions for further research.

## Data Availability Statement

The data that support the findings of this study are available from the corresponding author upon reasonable request.

## Ethics Statement

The studies involving human participants were reviewed and approved by Sapienza Ethics Committee. The patients/participants provided their written informed consent to participate in this study.

## Author Contributions

PV design of the work and critical revised the paper. CC, GR, and SB collected data. GR analyzed data. All authors contributed to draft the article.

## Conflict of Interest

The authors declare that the research was conducted in the absence of any commercial or financial relationships that could be construed as a potential conflict of interest.

## References

[1] World Health Organization. Coronavirus Disease 2019 (COVID-19): Situation Report, 30. World Health Organization (2020). Available online at: https://apps.who.int/iris/handle/10665/331119

[2] Mertens G Gerritsen L Duijndam S Salemink E Engelhard IM. Fear of the coronavirus (COVID-19): predictors in an online study conducted in March 2020. J Anxiety Disord. (2020) 74:102258. 10.1016/j.janxdis.2020.102258 32569905 PMC7286280

[3] Goyal K Chauhan P Chhikara K Gupta P Singh MP. Fear of COVID 2019: first suicidal case in India!. Asian J Psychiatr. (2020) 49:101989. 10.1016/j.ajp.2020.101989 32143142 PMC7130010

[4] Brandt CP Johnson KA Schmidt NB Zvolensky MJ. Main and interactive effects of emotion dysregulation and breath-holding duration in relation to panic-relevant fear and expectancies about anxiety-related sensations among adult daily smokers. J Anxiety Disord. (2012) 26:173–81. 10.1016/j.janxdis.2011.10.007 22119451 PMC3254822

[5] Steenmeijer A Kennis M. The relation between anxious personality traits and fear generalization in healthy subjects: a systematic review and meta-analysis. Neurosci Biobehav Rev. (2019) 107:320–8. 10.1016/j.neubiorev.2019.09.029 31557547

[6] Dymond S Dunsmoor JEJE Vervliet B Roche B Hermans D. Fear generalization in humans: systematic review and implications for anxiety disorder research. Behav Ther. (2015) 46:561–82. 10.1016/j.beth.2014.10.001 26459838

[7] Lopresto D Schipper P Homberg JR. Neural circuits and mechanisms involved in fear generalization: implications for the pathophysiology and treatment of posttraumatic stress disorder. Neurosci Biobehav Rev. (2016) 60:31–42. 10.1016/j.neubiorev.2015.10.009 26519776

[8] Mertens G Bouwman V Leer A Engelhard IM. Conceptual fear generalization gradients. Int J Psychophysiol. (2019). [Epub ahead of print]. 10.31234/osf.io/zwc2h 34606931

[9] Lanius RA Frewen PA Vermetten E Yehuda R. Fear conditioning and early life vulnerabilities: two distinct pathways of emotional dysregulation and brain dysfunction in PTSD. Eur J Psychotraumatol. (2010) 1. 10.3402/ejpt.v1i0.5467 22893793 PMC3401986

[10] Gritsenko V Skugarevsky O Konstantinov V Khamenka N Marinova T Reznik. COVID 19 fear, stress, anxiety, and substance use among Russian and Belarusian University students. Int J Ment Health Addict. (2020) 21:1–7. 10.1007/s11469-020-00330-z 32837418 PMC7241583

[11] Rossi R Socci V Talevi D Mensi S Niolu C Pacitti F. COVID-19 pandemic and lockdown measures impact on mental health among the general population in Italy. Front Psychiatry. (2020) 11:790. 10.3389/fpsyt.2020.00790 32848952 PMC7426501

[12] Masiero M Mazzocco K Harnois C Cropley M Pravettoni G. From individual to social trauma: sources of everyday trauma In Italy, The US and UK during the Covid-19 pandemic. J Trauma Dissociation. (2020) 21:513–9. 10.1080/15299732.2020.1787296 32654633

[13] Di Crosta A Palumbo R Marchetti D Ceccato I La Malva P Maiella R. Individual differences, economic stability, and fear of contagion as risk factors for PTSD symptoms in the COVID-19 emergency. Front Psychol. (2020) 11:567367. 10.3389/fpsyg.2020.567367 33013604 PMC7506146

[14] Xiong J Lipsitz O Nasri F Lui L Gill H Phan L. Impact of COVID-19 pandemic on mental health in the general population: a systematic review. J Affect Disord. (2020) 277:55–64. 10.1016/j.jad.2020.08.001 32799105 PMC7413844

[15] Neria Y Nandi A Galea S. Post-traumatic stress disorder following disasters: a systematic review. Psychol Med. (2008) 38:467–80. 10.1017/S0033291707001353 17803838 PMC4877688

[16] Breslau N Chase GA Anthony JC. Post-traumatic stress disorder following disasters: a systematic review. Psychol Med. (2002) 32:573–6. 10.1017/S0033291701004998 12102371

[17] Liu J Chen X Wang M Cheng L. Cognitive intervention on the flashback of traumatic event: based on the dual representation theory of PTSD. Int J Ment Health Promot. (2018) 20:75–82. 10.32604/IJMHP.2018.010857

[18] Hopwood CJ Wright AGC Ansell EB Pincus AL. The interpersonal core of personality pathology. J Pers Disord. (2013) 27:270–95. 10.1521/pedi.2013.27.3.270 23735037 PMC3675800

[19] Volkert J Gablonski TC Rabung S. Prevalence of personality disorders in the general adult population in Western countries: systematic review and meta-analysis. Br J Psychiatry. (2018) 213:709–15. 10.1192/bjp.2018.202 30261937

[20] Samuels J. Personality disorders: epidemiology and public health issues. Int Rev Psychiatry. (2011) 23:223–33. 10.3109/09540261.2011.588200 21923224

[21] Reis AM de Carvalho LF Elhai JD. Relationship between PTSD and pathological personality traits in context of disasters. Psychiatry Res. (2016) 241:91–7. 10.1016/j.psychres.2016.04.099 27156030

[22] Zanarini MC Frankenburg FR Dubo ED Sickel AE Trikha A Levin A. Axis I comorbidity of borderline personality disorder. J Psychiatry. (1998) 115:1733–9. 10.1176/ajp.155.12.1733 9842784

[23] Swartz M Blazer D George L Winfield I. Estimating the prevalence of borderline personality disorder in the community. J Pers Disord. (1990) 4:257–72. 10.1521/pedi.1990.4.3.257

[24] Shea MT Zlotnick C Weisberg RB. Commonality and specificity of personality disorder profiles in subjects with trauma histories. J Pers Disord. (1999) 13:199–210. 10.1521/pedi.1999.13.3.199 10498034

[25] Gunderson JG Sabo AN. The phenomenological and conceptual interface between borderline personality disorder and PTSD. Am J Psychiatry. (1993) 150:19–27. 10.1176/ajp.150.1.19 8417576

[26] Van Der Kolk BA Pelcovitz D Roth S Mandel FS McFarlane A Herman JL. Dissociation, somatization, and affect dysregulation: the complexity of adaptation to trauma. Am J Psychiatry. (1996) 153:83–93. 10.1176/ajp.153.7.83 8659645

[27] Zlotnick C Johnson DM Yen S Battle CL Sanislow CA Skodol AE. Clinical features and impairment in women with borderline personality disorder (BPD) with posttraumatic stress disorder (PTSD), BPD without PTSD, and other personality disorders with PTSD. J Nerv Ment Dis. (2003) 191:706–13. 10.1097/01.nmd.0000095122.29476.ff 14614337

[28] James LM Anders SL Peterson CK Engdahl BE Krueger RF Georgopoulos AP. DSM-5 personality traits discriminate between posttraumatic stress disorder and control groups. Exp Brain Res. (2015) 233:2021–8. 10.1007/s00221-015-4273-1 25862564

[29] Garcia R. Neurobiology of fear and specific phobias. Learn Memory. (2017) 24:462–71. 10.1101/lm.044115.116 28814472 PMC5580526

[30] LeDoux J. Il cervello Emotivo. Milano: Tr It Baldini&Castoldi (1998).

[31] LeDoux J. The emotional brain, fear, and the amygdala. Cell Mol Neurobiol. (2003) 23:727–38. 10.1023/A:1025048802629 14514027 PMC11530156

[32] Porges SW. The COVID-19 pandemic is a paradoxical challenge to our nervous system: a polyvagal perspective. Clin Neuropsychiatry. (2020) 17:135–8. 10.36131/CN20200220 PMC8629069 34908984

[33] Powers A Cross D Fani N Bradley B. PTSD, emotional dysregulation, and dissociative symptoms in a highly traumatized sample. J. Psychiatry. (2015) 61:144–79. 10.1016/j.jpsychires.2014.12.011 PMC4308496 25573648

[34] Serrano-Sevillano Á González-Ordi H Corbí-Gran B Vallejo-Pareja MÁ. Psychological characteristics of dissociation in general population. Clí*n Salud*. (2017) 28:101–6. 10.1016/j.clysa.2017.09.00316208166

[35] Briere J Scott C Weathers F. Peritraumatic and persistent dissociation in the presumed etiology of PTSD. Am J Psychiatry. (2005) 162:2295–301. 10.1176/appi.ajp.162.12.2295 16330593

[36] Tull M Barrett H McMillan ES Roemer L. A preliminary investigation of the relationship between emotion regulation difficulties and posttraumatic stress symptoms. Behav Ther. (2007) 38:303–13. 10.1016/j.beth.2006.10.001 17697854

[37] Gratz KL Roemer L. Multidimensional assessment of emotion regulation and dysregulation: development, factor structure, and initial validation of the difficulties in emotion regulation scale. J Psychopathol Behav Assess. (2004) 26:41–54. 10.1023/B:JOBA.0000007455.08539.94

[38] Weiss H Gratz KL Lavender JM. Factor structure and initial validation of a multidimensional measure of difficulties in the regulation of positive emotions: the DERS-Positive. Behav Modif. (2015) 39:431–53. 10.1177/0145445514566504 25576185 PMC4420643

[39] Gratz KL Roemer L. The relationship between emotion dysregulation and deliberate self-harm among female undergraduate students at an urban commuter university. Cogn Behav Ther. (2008) 37:14–25. 10.1080/16506070701819524 18365795

[40] Dimaggio G Popolo R Montano A Velotti P Perrini F Buonocore L. Emotion dysregulation, symptoms and interpersonal problems as independent predictors of a broad range of personality disorders in an outpatient sample. Psychol Psychother Theory Res Pract. (2017) 90:569–99. 10.1111/papt.12126 28585718

[41] Velotti P Garofalo C Callea A Bucks R Roberton T Daffern M. Exploring anger among offenders: the role of emotion dysregulation and alexithymia. Psychiatry Psychol Law. (2017) 28:128–38. 10.1080/13218719.2016.1164639 31983944 PMC6818369

[42] Gillespie S Garofalo C Velotti P. Emotion regulation, mindfulness, and alexithymia: specific or general impairments in sexual, violent, and homicide offenders? J Criminal Justice. (2018) 58:56–66. 10.1016/j.jcrimjus.2018.07.006

[43] Rogier G Velotti P. Narcissistic implications in gambling disorder: the mediating role of emotion dysregulation. J Gambl Stud. (2018) 34:1241–60. 10.1007/s10899-018-9759-x 29455443

[44] Rogier G Garofalo C Velotti P. Is emotional suppression always bad? A matter of flexibility and gender differences. Curr Psychol. (2019) 38:411–20. 10.1007/s12144-017-9623-7

[45] Garofalo C Velotti P Crocamo C Carrà G. Single and multiple clinical syndromes in incarcerated offenders: associations with dissociative experiences and emotionality. Int J Offend Ther Compar Criminol. (2018) 62:1300–16. 10.1177/0306624X16682325 27913716 PMC5858637

[46] Stiglmayr CE Ebner-Priemer UW Bretz J Behm R Mohse M Lammers CH. Dissociative symptoms are positively related to stress in borderline personality disorder. Acta Psychiatr Scand. (2008) 117:139–47. 10.1111/j.1600-0447.2007.01126.x 18028248

[47] Briere J. Dissociatives symptoms and trauma exposure: specificity, affect dysregulation, and posttraumatic stress. J Nerv Ment Dis. (2006) 194:78–82. 10.1097/01.nmd.0000198139.47371.54 16477184

[48] Brand BL Lanius RA. Chronic complex dissociative disorders and borderline personality disorder: disorders of emotion dysregulation?. Borderline Personality Disord Emot Dysregul. (2014) 1:13. 10.1186/2051-6673-1-13 26401297 PMC4579511

[49] Giromini L Velotti P de Campora G Bonalume L Zavattini GC. Cultural adaptation of the difficulties in emotion regulation scale: reliability and validity of an Italian version. J Clin Psychol. (2012) 68:989–1007. 10.1002/jclp.21876 22653763

[50] Carlson EB Putnam FW. An update on the dissociative experience scale. Dissociation. (1993) 6:16–27.

[51] Garofalo C Velotti P Zavattini GC Tommasi M Romanelli R Espirito Santo H. On the factor structure of the dissociative experiences scale: contribution with an Italian version of the DES-II. J Psychiatry Clin Psychol. (2015) 15:4–12. 10.15557/PiPK.2015.0001

[52] Krueger RF Derringer J Markon KE Watson D Skodol AE. Initial construction of a maladaptive personality trait model and inventory for DSM-5. Psychol Med. (2011) 42:1879–90. 10.1017/S0033291711002674 22153017 PMC3413381

[53] Fossati A Krueger RF Markonk E Borroni S Maffei C. Reliability and validity of the personality inventory for DSM-5 (Pid-5): predicting DSM-IV personality disorders and psychopathy in community-dwelling Italian adults. Assessment. (2013) 20:689–708. 10.1177/1073191113504984 24065702

[54] LeBeau R Mischel E Resnick H Kilpatrick D Friedman M Craske M. Dimensional assessment of posttraumatic stress disorder in DSM-5. Psychiatry Res. (2014) 218:143–7. 10.1016/j.psychres.2014.03.032 24746390

[55] Ahorsu DK Lin CY Imani V Saffari M Griffiths MD Pakpou AH. The fear of COVID-19 scale: development and initial validation. Int J Ment Health Addict. (2020) 1–9. [Epub ahead of print]. 10.1007/s11469-020-00270-8 32226353 PMC7100496

[56] Soraci P Ferrari A Abbiati FA Del Fante E De Pace R Urso A. Validation and psychometric evaluation of the Italian version of the fear of COVID-19 Scale. Int J Ment Health Addict. (2020). [Epub ahead of print]. 10.1007/s11469-020-00277-1 32372892 PMC7198091

[57] Nikopoulou VA Holeva V Parlapani E Karamouzi P Voitsidis P Porfyri GN. Mental health screening for COVID-19: a proposed cutoff score for the Greek version of the fear of COVID-19 scale (FCV-19S). Int J Ment Health Addict. (2020) 1–14. [Epub ahead of print]. 10.1007/s11469-020-00414-w 33199975 PMC7654349

[58] Browne MW Cudeck R. Alternative ways of assessing model fit. Sociol Methods Res. (1992) 21:230–58. 10.1177/0049124192021002005

[59] Kline RB. Promise and pitfalls of structural equation modeling in gifted research. In Thompson B Subotnik RF editors. Methodologies for Conducting Research on Giftedness. Washington, DC: American Psychological Association (2010). p. 147–169.

[60] Van Dijke A Hopman JAB Ford JD. Affect dysregulation, psychoform dissociation, and adult relational fears mediate the relationship between childhood trauma and complex posttraumatic stress disorder independent of the symptoms of borderline personality disorder. Eur J Psychotraumatol. (2018) 9:1400878. 10.1080/20008198.2017.1400878 29410773 PMC5795767

[61] Lanius R Vermetten E Loewenstein R Brand B Schmahl C Bremner JMD. Emotion modulation in PTSD: clinical and neurobiological evidence for a dissociative subtype. Ame J Psychiatry. (2010) 167:640–7. 10.1176/appi.ajp.2009.09081168 20360318 PMC3226703

[62] American Psychiatric Association DSM-5 Task Force. Diagnostic and Statistical Manual of Mental Disorders: DSM-5™, 5th ed. American Psychiatric Publishing, Inc. (2013). 10.1176/appi.books.9780890425596

[63] Lis A Parolin L Salcuni S Zennaro A. Rorschach comprehensive system data for a sample of 249 adult nonpatients from Italy. J Pers Assess. (2007) 89:S80–4. 10.1080/00223890701583275 18039175

[64] Barone L. Developmental protective and risk factors in borderline personality disorder: a study using the adult attachment interview. Attach Hum Dev. (2003) 5:64–77. 10.1080/1461673031000078634 12745829

[65] Goodwin RD Friedman HS. Health status and the five-factor personality traits in a nationally representative sample. J Health Psychol. (2006) 11:643–54. 10.1177/1359105306066610 16908463

[66] Janet P. L'Automatisme psychologiques : essay de psychologie expérimentale sur les formes inferieures de l'activite humaine. Paris, Felix Alcan Reprint : societe Pierre Janet/Payot, Paris (1989).

